# Genome-wide structural and evolutionary analysis of the P450 monooxygenase genes (P450ome) in the white rot fungus *Phanerochaete chrysosporium *: Evidence for gene duplications and extensive gene clustering

**DOI:** 10.1186/1471-2164-6-92

**Published:** 2005-06-14

**Authors:** Harshavardhan Doddapaneni, Ranajit Chakraborty, Jagjit S Yadav

**Affiliations:** 1 Environmental Genetics and Molecular Toxicology Division, Department of Environmental Health, University of Cincinnati College of Medicine, Cincinnati, OH 45267-0056, USA; 2 The Center for Genome Information, Department of Environmental Health, University of Cincinnati College of Medicine, Cincinnati, OH 45267-0056, USA

## Abstract

**Background:**

*Phanerochaete chrysosporium*, the model white rot basidiomycetous fungus, has the extraordinary ability to mineralize (to CO_2_) lignin and detoxify a variety of chemical pollutants. Its cytochrome P450 monooxygenases have recently been implied in several of these biotransformations. Our initial P450 cloning efforts in *P. chrysosporium *and its subsequent whole genome sequencing have revealed an extraordinary P450 repertoire ("P450ome") containing at least 150 P450 genes with yet unknown function. In order to understand the functional diversity and the evolutionary mechanisms and significance of these hemeproteins, here we report a genome-wide structural and evolutionary analysis of the P450ome of this fungus.

**Results:**

Our analysis showed that *P. chrysosporium *P450ome could be classified into 12 families and 23 sub-families and is characterized by the presence of multigene families. A genome-level structural analysis revealed 16 organizationally homogeneous and heterogeneous clusters of tandem P450 genes. Analysis of our cloned cDNAs revealed structurally conserved characteristics (intron numbers and locations, and functional domains) among members of the two representative multigene P450 families CYP63 and CYP505 (P450foxy). Considering the unusually complex structural features of the P450 genes in this genome, including microexons (2–10 aa) and frequent small introns (45–55 bp), alternative splicing, as experimentally observed for CYP63, may be a more widespread event in the P450ome of this fungus. Clan-level phylogenetic comparison revealed that *P. chrysosporium *P450 families fall under 11 fungal clans and the majority of these multigene families appear to have evolved locally in this genome from their respective progenitor genes, as a result of extensive gene duplications and rearrangements.

**Conclusion:**

*P. chrysosporium *P450ome, the largest known todate among fungi, is characterized by tandem gene clusters and multigene families. This enormous P450 gene diversity has evolved by extensive gene duplications and intragenomic recombinations of the progenitor genes presumably to meet the exceptionally high metabolic demand of this biodegradative group of basidiomycetous fungi in ecological niches. In this context, alternative splicing appears to further contribute to the evolution of functional diversity of the P450ome in this fungus. The evolved P450 diversity is consistent with the known vast biotransformation potential of *P. chrysosporium*. The presented analysis will help design future P450 functional studies to understand the underlying mechanisms of secondary metabolism and oxidative biotransformation pathways in this model white rot fungus.

## Background

The cytochrome P450 monooxygenases ("P450s") constitute a large superfamily of heme-thiolate proteins widely distributed in different life forms including prokaryotes (archaea, bacteria), lower eukaryotes (fungi, insects), and higher eukaryotes (plants and animals). P450s play an important role in the metabolism of a wide variety of endogenous and xenobiotic compounds. The current P450 nomenclature [1] is based on divergent evolution of the P450 superfamily. On the basis of sequence homology, all P450s can be categorized into two main classes, B ('bacterial') and E ('eukaryotic') [2]. Bacterial P450s with three component systems [an FAD-containing flavoprotein (NADPH or NADH-dependent reductase), an iron-sulphur protein, and the P450 hemeprotein] and the fungal P450nor (CYP55) [3] belong to the 'B'-class. All the other known P450s from distinct systems, including eukaryotic P450s and bacterial P450s such as P450BM3 (CYP102) from *Bacillus megaterium *[4], belong to the 'E'-class. The eukaryotic microsomal P450 system contains two components, the NADPH:P450 oxidoreductase (POR), a flavoprotein containing both FAD and FMN, and the P450 monooxygenase containing the heme domain. The prokaryotic (bacterial) soluble P450 monooxygenase P450BM3 (CYP102) exists as a single protein with both heme and flavin functional domains. Typically, the bacterial P450s are soluble and shorter (approximately 400 amino acids) in length, whereas the eukaryotic P450s average around 500–600 amino acids or larger and are membrane-bound. The amino acid (aa) sequence of these P450 monooxygenase proteins is extremely diverse, with levels of identity as low as 16% in some cases, but their structural fold has remained the same throughout evolution. The existing data suggest that divergence of the P450 superfamily into B and E classes, and further divergence into stable P450 groups within the E class, is very ancient and had occurred before the appearance of eukaryotes [5]. From the phylogenetic classification point of view, families have been identified based primarily on amino acid sequence similarity, with less than 40% similarity defining a family and 40–55% similarity defining a sub-family. Recently, the concept of "Clan" which represents higher order grouping of P450 families is gaining wider acceptance in the P450 community [6,7].

Among the different phyla, plants have the highest number of P450 sequences, followed by fungi. So far, more than 380 fungal P450s have been identified and the number is increasing with the sequencing of new fungal genomes . In comparison to the yeast forms (Saccharomyces and Candida), the higher order fungi have a larger number of P450 sequences in their genomes, with 41 P450 genes predicted in *Neurospora crassa *, 111 in *Aspergillus spp *., 123 in *Magnaporthe grisea *, 110 in *Fusarium graminearum *and about 150 in *Phanerochaete chrysosporium. *Assuming the possibility that the present day P450s have evolved from a single ancestor P450 gene, the large disparity in the P450 gene count among the different phyla and genera is reflective of the differences in metabolic demand. According to Nelson [8], while plants have invested heavily on biochemistry for development of survival strategies thereby driving the P450s to expand rapidly, animals have invested on development of higher order sensory systems and hence carry comparatively fewer P450s. Fungi, which resemble plants in their sedentary habitat, appear to have driven P450s to diversify rapidly.

*Phanerochaete chrysosporium *, the model white rot fungus, has the extraordinary ability to degrade and mineralize (to CO_2_) lignin, the earth's most abundant aromatic polymer, and a wide range of toxic chemical pollutants. Lignin biodegradation occurs under nutrient-limited conditions when the fungus enters secondary metabolism. Only white rot fungi are known to possess the ability to completely degrade lignin [9],[10],[11]. The working hypothesis is that initial depolymerization of the lignin by extracellular peroxidases releases chemical compounds that are internalized and further degraded by diverse intracellular enzymes, including P450 monooxygenases. Further, several studies have shown that this fungus can degrade and mineralize a broad spectrum of aromatic, alicyclic, and aliphatic chemical pollutants under both nutrient-limited (ligninolytic) and nutrient-sufficient (non-ligninolytic) conditions. Lately, P450 monooxygenases have been shown to be involved in several of these biotransformations and this has led to an increasing interest in the characterization of cytochrome P450 systems in this model white rot fungus.

Initial cloning efforts from this laboratory identified the first complete P450 system genes in *P. chrysosporium*. These efforts led to the isolation [12] and functional characterization [14,15,17] of three full-length P450 genes *pc*-1, *pc*-2 and *pc*-3 that were assigned to CYP63 family, and the P450 oxidoreductase gene (*POR *) that is responsible for electron transfer to the multiple P450 monooxygenases [16]. Subsequent whole genome sequencing by the Joint Genome Institute (JGI) of the US Department of Energy (US-DOE) has led to the identification of a large P450 contingent (P450ome) in *P. chrysosporium *[13]. The P450 genes constitute nearly 1% of the coding sequences in the genome of this basidiomycetous fungus [13]. Of the nearly 150 P450 monooxygenase genes identified in *P. chrysosporium *genome, 108 have been assembled full-length and tentatively annotated based on general overall sequence homology analysis [13]. To date, this is the highest number of P450s identified in any fungus, which appears to be one of the major underlying factors for its extraordinary catalytic potential. Understanding the structural and evolutionary aspects of such large family of P450s in conjunction with their physiological characteristics will help understand the functional versatility of this fungus. This study reports a detailed genome-wide structural and phylogenetic analysis of the P450ome of *P. chrysosporium *, coupled with cloning and characterization of new cDNAs for selected multigene P450 families, with a broader aim to facilitate the classification/nomenclature and to understand the functional diversity and evolution of the P450s in this wood-rotting model white rot fungus.

## Results and Discussion

### I. Structural analysis and characterization of *P. chrysosporium *P450ome

#### P450ome of *P. chrysosporium*

**Figure 1:**
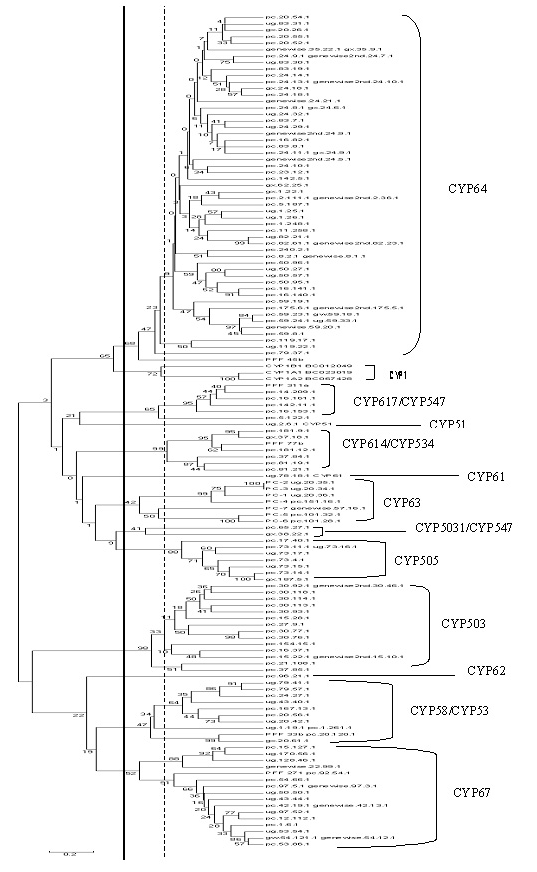
**P450ome of the white rot basidiomycete *Phanerochaete chrysosporium *. **The UPGMA tree is based on 126 full-length or near full-length P450s of *P. chrysosporium *P450ome and uses the three human CYP1 proteins (1A1, 1A2, and 1B1) for comparison. There are 12 families demarcated using the solid vertical line and 23 sub-families differentiated using the dashed vertical line.

Our phylogenetic analysis coupled with the standard sequence homology criterion for P450 nomenclature revealed that *P. chrysosporium *P450s fall into 12 families and 23 sub-families (Figure 1). This family and sub-family classification is based on the amino acid sequence similarity using the existing criteria of less than 40% similarity defining a family and less than 55% similarity defining a sub-family as followed by the International P450 Nomenclature Committee. Earlier we had reported an initial phylogenetic grouping of 163 predicted P450 sequences (including even partial fragments) into 26 clusters based on overall general homology criterion [15]. Here we report the phylogenetic grouping of 126 P450 genes in 12 families and 23 sub-families using the P450 family/sub-family homology-based criterion. To eliminate the possibility of bias in grouping, only full-length or near full-length P450 sequences with more than 300 aa residues were used for constructing the tree in this study. Of the 12 families, the International P450 Nomenclature Committee so far has named only three families including the highly conserved families CYP51 and CYP61, and the newly designated *P. chrysosporium *family CYP63. Nevertheless, based on the < 40% homology criterion, nine other families, each with varying degree of similarity to the existing one or more P450 families, were identifiable and were arbitrarily designated as follows: CYP58/53, CYP62, CYP64, CYP67, CYP503, CYP505, CYP5031/547, CYP614/534, CYP617/CYP547. As compared to the recently reported analysis [13], we have added 18 P450 genes grouped as three new clusters in the tree. Individual genes from these clusters, when compared to the P450 sequences available at the P450 BLAST server , showed highest similarity to the P450 families CYP617/547, CYP614/534 and CYP5031/547, respectively. These three sets of CYP names thus represent the families newly added to our phylogenetic tree (Figure 1).

Among the 12 P450 families, the CYP64 family has the highest number (more than fifty) of member genes, followed by CYP67 (sixteen), CYP503 (fourteen), CYP58/53 (ten), CYP63 (seven), CYP505 (seven), CYP614/534 (seven), CYP617/547 (six), CYP5031/CYP547 (two), whereas P450 families CYP51, CYP61 and CYP62 consist of only one member gene each.

The International Nomenclature Committee on cytochrome P450s has been annotating the new P450 sequences on the basis of the existing primary amino acid sequence similarity criteria. However, there have been instances where the 40% similarity rule in defining a family has been relaxed, such as in naming the mammalian CYP4 family [7]. Simultaneously, the usage of CYP "Clans" which are higher-order clusters of related families is gaining consensus [6,7]. In view of this, a clan-based classification of the P450 families in *P. chrysosporium *is presented and discussed in subsequent sections, with an aim to understand their evolution and functional significance.

#### Cloning of new P450 cDNAs

Our initial studies on cloning of cDNAs for the first cloned P450 gene *pc*-1 [12] and the related gene *pc*-3 [17], both belonging to CYP63 family, indicated a complex structural organization of P450 genes in *P. chrysosporium*. In an effort to further understand the structural organization of P450 genes in this system, the following additional cDNA sequences for the other CYP63 genes were isolated in the present study: *pc*-2 (1842 bp), *pc*-4 (430 bp), *pc*-5 (324 bp) and *pc*-6 (330 bp). The isolated cDNA sequences (full-length and partial) were then compared with the corresponding predicted coding sequences in the genome. This gene/cDNA analysis helped understand the gene features of the first multigene P450 family (CYP63) identified in this organism [12]. The analysis also helped identify an additional gene, *pc*-7, groupable under this family. Furthermore, we isolated a cDNA sequence representing the C-terminus half (the reductase domain) of one of the fused P450foxy-like genes, *pc-foxy*1. The cloned cDNA (681 bp), that spans 747 bp of the corresponding genomic region, helped identify the structural features of the P450foxy gene family (CYP505) of the P450ome.

#### Gene features (introns, exons and deduced coding regions) of the *P. chrysosporium *P450s

##### i). CYP63 family

**Figure 2:**
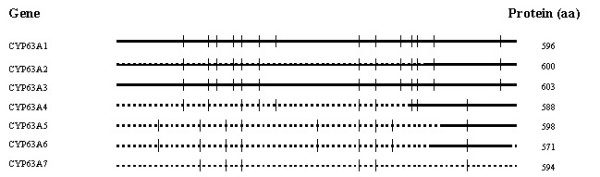
**Gene organization of the seven members of CYP63 family of P450s in the white rot fungus *P. chrysosporium. ***Horizontal lines represent the predicted coding region (exons) and the vertical lines indicate intron locations. Full-length cDNAs isolated for *pc*-1, *pc*-2 and *pc*-3, and partial cDNAs isolated for *pc*-4 (430 bp), *pc*-5 (324 bp) and *pc*-6 (330 bp), are shown by the solid horizontal lines.

**Figure 3:**
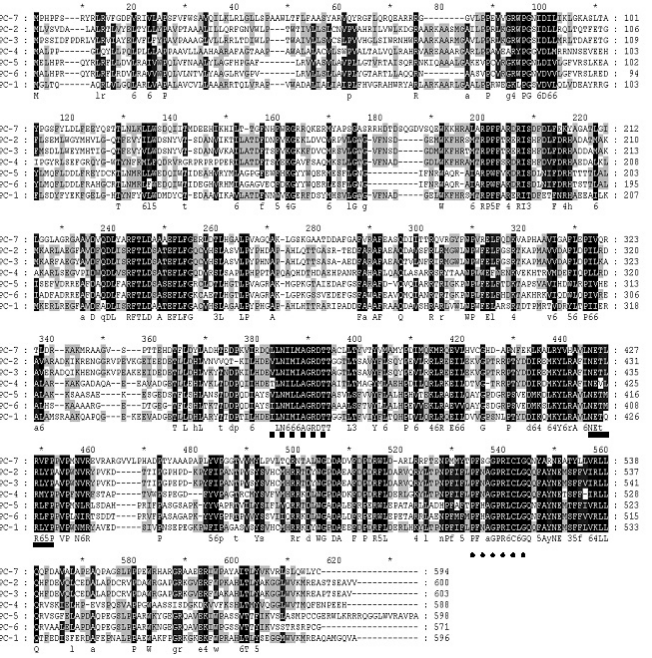
**Alignment of amino acid sequences of the CYP63 family P450s in *P. chrysosporium*. **The seven member proteins PC-1 through PC-7 were aligned using the GeneDoc program version 2.6.002 in conserved format. Relatively conserved heme-binding (HR2) region, helix-I and helix-K for the seven sequences are underlined with square-dotted line, solid line and round-dotted line, respectively. The alignment shading is based on the physicochemical properties group analysis, wherein each column in the alignment is assigned to one of the twelve predefined groups of physicochemical properties. These twelve groups represent three overlapping hierarchies of size, the electrical charge for the polar amino acids, and the aromaticity for non-polar amino acids.

The cloned CYP63 cDNA sequences revealed that the member genes carry 6–14 introns with conserved GT/AG splice junctions and encode 571 to 603 aa proteins. The deduced proteins contain transmembrane domains in their N-terminus region centered around 21–57 aa residues with a matrix score of more than 1000 as determined by TMpred analysis, indicating that these proteins are membrane bound. The seven CYP63 members (*pc*-1 through *pc*-7) are groupable into three sub-families, CYP63A (*pc*-1 through *pc*-4), CYP63B *(pc*-5 and *pc*-6), and CYP63C *(pc*-7). The three CYP63A member genes *pc*-1 (CY63A1), *pc*-2 (CY63A2) and *pc*-3 (CY63A3) are tandemly linked in that order with 322 bp and 469 bp intergenic regions. Their intron organization (number and position) and the number of amino acids encoded are nearly the same (Figure 2), suggesting that the three genes are paralogous genes and originated by tandem duplication. The typical P450 motifs including heme-binding region (HR2), helix-I, and helix-K showed high sequence conservation among the seven CYP63 member proteins (Figure 3).

##### ii). P450foxy gene family (CYP505)

**Figure 4:**
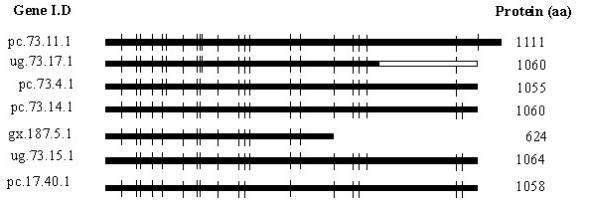
**Gene organization of the seven fused oxidoreductase-P450 (P450foxy-like) proteins. **Horizontal bars represent the predicted coding regions (exons) and the vertical lines indicate intron locations. The unfilled part of the bar represents the cloned cDNA portion of the gene ug.73.17.1, whereas the solid bars indicate the predicted cDNAs.

The *P. chrysosporium *whole genome sequence revealed seven fused oxidoreductase-P450 proteins ("P450foxy" proteins), of which 5 are located on a 43 kb stretch of the genome scaffold 73. The process of deducing the amino acid sequences for these genes was guided in part by the *pc-foxy*1 cDNA cloned in this study, in conjunction with the gene characteristics of the known P450foxy sequence of *Fusarium oxysporum *[18], and the earlier reported P450 splicing pattern in *P. chrysosporium *[12]. Analysis of the P450foxy genes revealed 17 to 22 introns, of which 11 are conserved in all the seven member genes (Figure 4). The cloned *pc-foxy*1 cDNA fragment (681 bp) contained splicing site for a single Type-II intron (66 bp) with defined GT/AG splice junction. The reductase portion of the cloned *pc-foxy*1 cDNA contained two FAD and three NADPH functional domains and showed deduced aa homology to the distal region of the P450 oxidoreductase (POR) protein of this organism. The full-length (736 aa) functional POR earlier reported for *P. chrysosporium *has multiple domains, including 3 FMN, 3 FAD and 3 NADPH domains [16]. However, except for the proximal FAD domain, the degree of motif conservation was poor between the two reductase sequences (*pc-foxy*1 reductase component and the POR), suggesting that the reductase component of the P450foxy fusion genes has an independent descent and is not duplicated from the native POR gene.

The five P450foxy member genes on genome scaffold 73 show divergent transcription, with the three member genes (ug.73.17.1, pc.73.4.1 and pc.73.14.1) transcribing from the same strand, whereas the other two genes (ug.73.15.1 and ug.73.16.1) transcribing from the opposite strand of DNA. The deduced P450foxy proteins containing 624–1111 amino acid residues show high aa similarity (> 55%) among them and hence can be classified under the same sub-family. The general domain architecture of these proteins is the same as that of the earlier reported P450foxy protein from *F. oxysporum *[18], with an N-terminal P450 segment and a C-terminal reductase segment. All seven proteins have a full-length P450 segment with conserved P450 signature sequences. However, the reductase segment is full-length in only 6 of the seven members and is truncated in the member gene gx.187.5.1. These six member proteins contain a transmembrane domain, centered around 908–920 aa positions with a matrix score of more than 1000, as detected by TMpred analysis. The truncated member contains a transmembrane domain centered on amino acid residue 570. The above analysis points to the membrane-bound nature of these fused proteins similar to the homologous *F. oxysporum *protein and consistent with the membrane-bound nature of most of the eukaryotic P450 and POR proteins.

It is unusual that *P. chrysosporium *has only one P450 oxidoreductase (POR) gene to cater to such a large contingent of P450 monooxygenases for the electron supply, with the exception of P450foxy proteins (see below). This suggests a more significant role of alternate electron transfer proteins such as cytochromes b5 and b5 reductase in this organism; it will, however, require further functional characterization to prove this assumption. The P450foxy proteins appear to be self sufficient in their electron transfer mechanism considering the presence of a complete reductase partner (with all needed functional domains) as a part of the fusion protein. Such electron transfer function of the reductase component was experimentally demonstrated in the first known P450foxy fusion protein of the ascomycete fungus *Fusarium oxysporum *[18].

##### iii). Gene features of other P450ome genes

Among the other P450 families in the P450ome, the CYP64 family has the most genes (54), with member genes carrying 5–12 introns of size 21–268 bp, and encoding 350–534 aa size proteins. Detailed gene features of the other individual P450 families of the *P. chrysosporium *P450ome are listed in Table 1.

Typically, all the full-length assembled P450 genes were found to carry multiple exons frequently interrupted by small introns. In this analysis, we observed a relatively narrow size range for introns (21 to 620 bp) as compared to the exons; the shortest exon is 6 bp (2 aa) and the longest is 1048 bp (Table 1). Nearly half of the P450 genes show small exons (upto 67 bp), with 41 genes carrying 29 bp or shorter exons (Table 1). Interestingly, the P450ome genes contain microexons (encoding 2–10 aa) that are spread genome-wide and are not restricted to any particular P450 family. Such ubiquitous occurrence of the microexons in *P. chrysosporium *P450 genes points to their potential role in conferring functional diversity and emphasizes the fact that the functional divergence is not merely a product of gene duplication/translocation. In comparison to the P450 genes, the intron size range in the most intensively characterized peroxidase gene family of this organism is 49–78 bp, with 49 to 78 bp for the lignin peroxidase (LiP) sub-family and 50 to 72 bp for the manganese-dependent peroxidase (MnP) sub-family. Furthermore, as revealed by the whole genome sequencing, the presence of relatively small introns (average 117 bp) appears typical of this genome as compared to the higher eukaryotic genomes [13].

#### P450 gene clustering and tandem genes

The *P. chrysosporium *genome shows a total of 16 P450 gene clusters, with up to 11 member genes in a cluster. Of the 16 clusters, three clusters are located on scaffold 30, two each on scaffolds 20, 24, and 73, and one each on scaffolds 1, 16, 50, 53 59, 79 and 97. For convenience, the clusters have been assigned numbers 1 through 16 in the order they appear on the individual genomic scaffolds. Among these, cluster-5 has the highest number of tandem genes (eleven) followed by cluster-3 (five) and cluster-6 (four) (Table 2). The earlier cloned tandem CYP63 genes *pc*-1, *pc*-2 and *pc*-3 represent cluster-4.

There is a high sequence similarity (up to 84%) among the members of a given cluster and the cluster members fall under the same P450 family on the phylogenetic tree (Figure 1; Table 2). The number of introns and exons and their relative positions in the member genes of a cluster are conserved in 10 of the 16 clusters (Table 2). Two distinct patterns exist for the intron organization (number and position) within each cluster. In the case of small gene clusters (2–3 member genes), the number and relative position of introns as well as the size of the exons are conserved among the members, whereas the larger gene clusters (> 3 member genes) show varying degrees of dissimilarity in these characteristics. While the conserved gene characteristics suggest more recent duplications, the dissimilarity suggests either more distant duplication events or translocation events. Therefore, the *P. chrysosporium *genome shows both homogeneous and heterogeneous clusters of tandem genes. Tandem duplicates (paralogous genes), which initially are structurally and functionally identical, diverge with time due to mutations or translocations. It is plausible that one copy retains the original function, while the second copy either acquires a new function that is selected to meet the increased metabolic demand or gets deleted from the genome. In this context, it is noteworthy that member genes of cluster-4 (*pc*-1, *pc*-2 and *pc*-3), though closely spaced and structurally highly homogeneous, showed non-coordinate regulation of transcription and varying substrate inducibility [14,17]. While such substrate diversity among the members of tandemly linked P450 genes has been observed in the yeast *Candida maltosa *[19], this observation is new in the context of filamentous fungi.

Analysis of spatial organization of the P450 gene clusters in the *P. chrysosporium *genome revealed a variable pattern. For instance, the two P450 clusters on scaffold-20 are spread over a 220 kb genomic region separated by more than 200 kb, whereas the gene clusters on other scaffolds are more closely spaced. On scaffold-24, the clusters 5 and 6 are spread over a 62 kb of genomic region and are separated by less than 10 kb genomic region. Similarly, clusters 7 to 9 on scaffold-30 are spread over an 80 kb region with a 27–28 kb gap between them. The two clusters on scaffold-73 are separated by less than 10 kb DNA and are spread over a 30 kb genomic region. This analysis constitutes the first report on P450 clustering and spatial organization in filamentous fungi. Nevertheless, in fungi, it has been frequently observed that the genes coding for enzymes involved in secondary metabolism, such as those involved in the synthesis of ergot alkaloids [20], HC-toxin [21], and mycotoxins such as sterigmatocystin and aflatoxins [22,23], are heterologous clusters (containing P450 and other metabolic genes). Clustering of secondary metabolic genes has been proposed to favor their survival and dispersal, at least in part, via horizontal gene transfer [23,24]. The close association seen in some of the *P. chrysosporium *gene clusters might point to their co-ordinated regulation as observed in the case of the above secondary metabolic gene clusters in different fungi. However, experimental evidence is needed to extrapolate this assumption to the fungal P450 clusters. Our initial transcription-based analysis demonstrated lack of such co-ordinated regulation among the tandem CYP63 genes of cluster-4 in *P. chrysosporium *[14].

#### Alternative splicing and functional diversity

Alternative splicing is an important mechanism for regulation of gene expression, which expands the coding capacity of a single gene to allow production of different protein isoforms, often with diverse functions [25]. More than 50% of human genes are alternatively spliced [26]. Reports in fungi on alternative splicing are few [12,27], in comparison to humans where this mechanism has been well documented. We have experimentally identified two splice variants of the first characterized P450 gene *pc*-1 (CYP63A1) from *P. chrysosporium *[12] and predicted more such variants based on the *in silico *analysis. Although splice variants have not been identified so far in the other two tandemly arranged members *pc*-2 and *pc*-3 of this cluster, their similar gene organization (typically marked by multiple introns and exons as small as 4 to 10 aa length) suggests the existence of splice variants. Further, while validating our custom 70-mer microarray analysis data [15] on P450 gene transcription and induction using RT-PCR (wherein gene specific primers were chosen from different locations on the gene), we observed that the transcript quantification for a given sample in some cases varied with the location of the primer chosen (Unpublished data). This observed variation points to the existence of alternative splice variants. A recently proposed system of nomenclature for such splice variants suggests that the transcript name should include the exon involved in the splicing event [7]. It is noteworthy that nearly one-third of the *P. chrysosporium *P450ome shows microexons, which are likely candidates that promote alternative splicing events during transcription. Such alternative splicing helps increase the diversity of the transcriptome, and is likely to significantly contribute to the metabolic diversity of this organism.

### II. Evolutionary analysis of *P. chrysosporium *P450ome

#### Fungal P450 clans in *P. chrysosporium*

Finding meaningful associations and evolutionary relationships among members of the rapidly expanding superfamily of the P450 proteins at the species level is becoming a challenge using the existing family level classification. For instance, the CYP6 family that is exclusive to insects forms a close cluster with the CYP3 and CYP5 families from mammals, CYP30 from clams, and CYP25 from *C. elegans *on the phylogenetic tree, indicating that these five families probably have evolved from a common ancestor with similar function before the deuterostome-protostome split [8]. However, this is not reflected in the family names, as the family classification is based on an arbitrary 40% amino acid similarity criterion. To explain such higher order groupings, the term "Clan" was recently introduced [6]. Typically, a clan represents a cluster of P450 families across species, grouped based on relationships that are beyond the family designations. There are 9 clans in vertebrates and 10 in plants.

A detailed phylogenetic analysis was carried out to understand the evolutionary relationship of *P. chrysosporium *P450 gene families and their relatedness with other fungal clans. In lower forms of fungi (yeasts), 4 P450 families (CYP51, 52, 53 and 61) have been characterized, of which CYP51 and 61 are conserved. In higher forms of fungi (filamentous fungi), 13 P450 families (CYP51, 53, 61, 65, 68, 505, 531, 532, 537, 539, 540, 548, and 552) are common as exemplified by the hitherto sequenced genomes of four ascomycetous fungal species: *Neurospora crassa *, *Magnoporthe grisea *, *Fusarium graminearum *and *Aspergillus nidulans *. Interestingly, based on homology analysis, the P450 families CYP51, 53, 61, and 505 are also present in *P. chrysosporium *(a basidiomycetous fungus) albeit with a widely varying degree of similarity. Further, clan level comparisons revealed that 12 *P. chrysosporium *P450 families (Figure 1) have resemblances in 11 fungal P450 clans and show varying degrees of structural similarities to the P450 genes from different ascomycetous fungi such as *Aspergillus *and *Fusarium *, suggesting that *P. chrysosporium *has acquired these P450 families as a part of vertical descent from a common ancestor followed by further diversification. It will be interesting to compare the hitherto uncharacterized zygomycetous P450ome to arrive at the ancestral P450ome that led to the current P450 diversity among these three major fungal groups (ascomycetes, zygomycetes and basidiomycetes).

##### CYP51 clan

**Figure 5:**
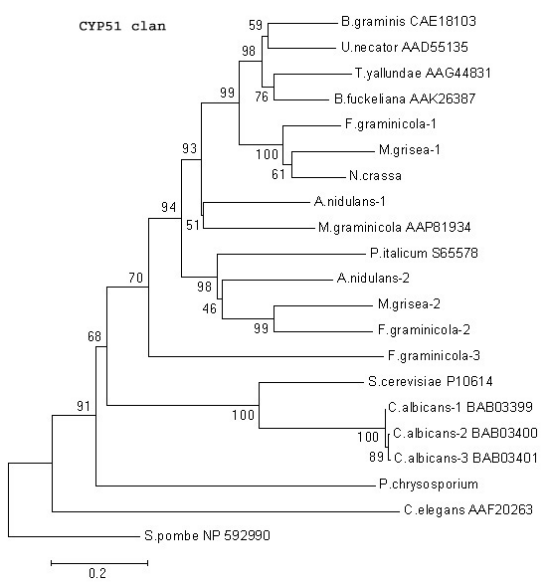
**Minimal evolution tree of the fungal CYP51 clan**. The fungal species included are *Aspergillus nidulans; Blumeria graminis, Botryotinia fuckeliana, Candida albicans, Cryptococcus neoformans, Cunninghamella elegans, Fusarium graminicola, Meloidogyne graminicola, Magnaporthe grisea, Neurospora crassa, Penicillium italicum, Phanerochaete chrysosporium, Saccharomyces cerevisiae, Schizosaccharomyces pombe, Tapesia yallundae, Uncinula necator*, and *Ustilago maydis. *

One of the highly conserved and functionally well-characterized P450 gene families in fungi is the CYP51 family that encodes the cell wall ergosterol biosynthesis reaction, lanosterol 14-alpha demethylation in yeasts or eburicol 14-alpha demethylation in filamentous fungi, a target step for antifungal azole compounds. The current state of knowledge on P450 evolution in eukaryotes points to CYP51 as the ancestral P450, which is believed to have led to the evolution of all the present day P450 families [8]. In comparison to some of the ascomycetous fungi, which carry multiple CYP51 genes, there is a single CYP51 gene (located on scaffold-2) in *P. chrysosproium *that codes for a 549 aa protein. The CYP51 minimal evolution tree, based on the available CYP51s from 17 fungal species (Figure 5), depicts that basidiomycete CYP51s (including *P. chrysosporium *CYP51) form the closest association with CYP51s from the ascomycetous fungi as compared to those from the zygomycetous fungus *Cunninghamella elegans *and the hemi-ascomycetous fungus (budding yeast). Considering the fact that all known fungi except ascomycetes have a single CYP51 gene in their genome (Figure 5), it appears that the ascomycetous fungi required diversification of their CYP51 gene. This intragenome diversification of CYP51 in ascomycetes, such as in the genomes of *A. nidulans *, *M. grisea *, and *F. graminearum *, is reflected by high bootstrap values separating their multiple CYP51s. Likewise, analysis of the recently completed plant genomes has shown that the rice genome contains ten functional CYP51 genes and two pseudogenes [28], and that Arabidopsis has two CYP51 genes, in contrast to the popular belief that a single CYP51 gene exists in all phyla. The presence of multiple CYP51s might indicate their involvement beyond the sterol biosynthesis to take up new function(s) for the organism's survival. Since the ascomycete species that carry multiple CYP51s are parasitic in nature, multiple CYP51s/variants might have been acquired to give these species an edge in host-pathogen interaction or survival against antifungal treatment [29], unlike in saprophytic fungi such as *P. chrysosporium*.

##### CYP 61 clan

**Figure 6:**
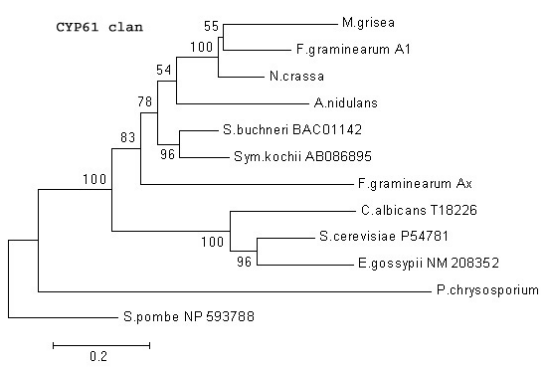
**Minimal evolution tree of the fungal CYP61 clan. **The fungal species included are *Aspergillus nidulans; Candida albicans, Eremothecium gossypii, Fusarium graminearum, Magnaporthe grisea, Neurospora crassa, Phanerochaete chrysosporium, Saccharomyces cerevisiae, Schizosaccharomyces pombe, Symbiotaphrina buchneri*, and *Symbiotaphrina kochii *.

CYP61 codes for sterol 22 desaturase in *Saccharomyces cerevisiae *, which is involved in later stages of the ergosterol pathway in metabolizing Ergosta-5,7,24(28)-trienol to Ergosta-5,7,22,24(28)-tetraenol by introducing a C-22(23) double bond in the sterol side chain [30]. Since this gene acts late in the ergosterol pathway, it is considered to have evolved as a result of duplication and diversification of the CYP51 gene [8]. The *P. chrysosporium *genome contains one CYP61 gene. However, unlike its CYP51 (Figure 5), CYP61 of *P. chrysosporium *is phylogenetically as distant from euascomycetous CYP61s as it is from the hemi-ascomycetous CYP51 (Figure 6). These analyses also show diversification of CYP61 within the ascomycetous group at least in 2 subclusters (with high bootstrap values) that coincide with small genomes (yeast-like) versus larger genomes (filamentous fungi).

##### CYP52 Clan

**Figure 7:**
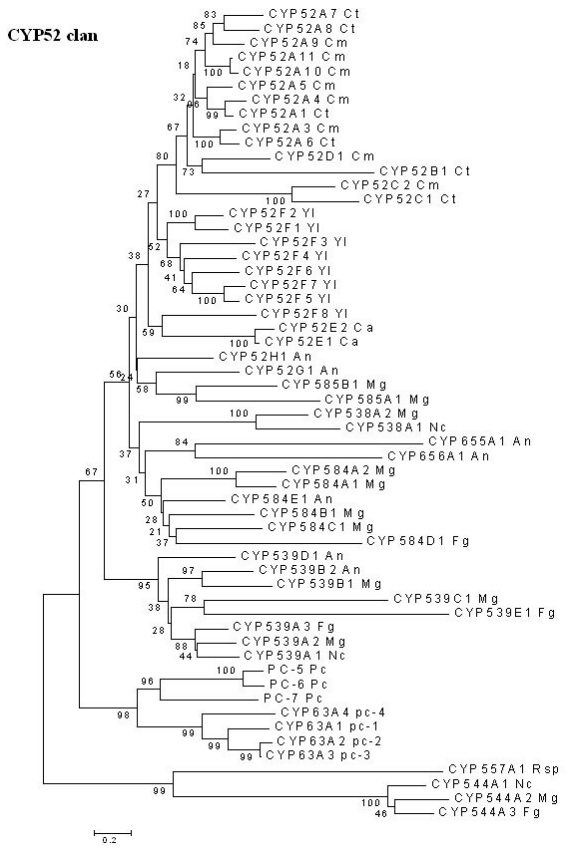
**Minimal evolution tree of the fungal CYP52 clan. **Abbreviations: An-*Aspergillus nidulans *; Ca-*Candida apicola *; Cm-*Candida maltosa *; Ct-*Candida tropicalis *; Fg-*Fusarium graminearum *; Mg-*Magnaporthe grisea *; Nc-*Neurospora crassa *;Pc-*Phanerochaete chrysosporium *; Rsp-*Rhodotorula *sp; Y*l *-*Yarrowia lipolytica *.

The CYP52 family of P450 proteins was originally identified in *Candida *species [31,32] with a role in carrying out terminal hydroxylation of n-alkanes and ω-hydroxylation of fatty acids. The varying substrate specificities of these genes have given this yeast its ability to modify a range of n-alkanes. Later, homologous genes have been identified in other yeasts [33,34] and filamentous fungal species (Figure 7). The earlier discussed CYP63 family of *P. chrysosporium*, which consists of seven members, *pc-*1 through *pc-*7, shows close architectural resemblance to the CYP52 family of yeasts and thus can be classified under the same clan. However, as evident from the minimal evolution tree (Figure 7), the white rot CYP63 members form a separate cluster on the tree from the CYP52 family members of the ascomycetous fungi and other CYP52-related fungal P450 families. Furthermore, despite high structural similarity among them, the individual member genes of the CYP63 cluster are separated by high bootstrap values, indicating significant divergence among them. This is corroborated by our experimental studies on substrate inducibility of the three tandemly linked CYP63 members *pc-*1, *pc-*2, and *pc-*3; the expression of these linked P450s was differentially induced (both in qualitative and quantitative terms) in response to linear alkanes and substituted alkanes. Moreover, their substrate-specificity seems to extend beyond alkanes/substituted alkanes as they also showed induction in the presence of various aromatics, including the polycyclic aromatic compounds such as PAHs [14,15]. These and other experimentally generated data on their differential physiological regulation [14] coupled with the presented phylogenetic analysis point to the evolution of the CYP63 family to acquire diverse functional roles while retaining its original alkane-degrading ability. While the n-alkanes are hydroxylated for assimilation (as carbon source) by *Candida *species, the P450 hydroxylation of alkanes by *P. chrysosporium *(which does not use them as sole carbon source) appears to be a co-metabolic activity required for catalyzing aliphatic oxidations during the natural lignin degradation process. This is further supported by the fact that there are structural resemblances between the inducer compounds (alkanes and aromatics) and the substructures present in its natural substrate lignin.

##### CYP64 clan

**Figure 8:**
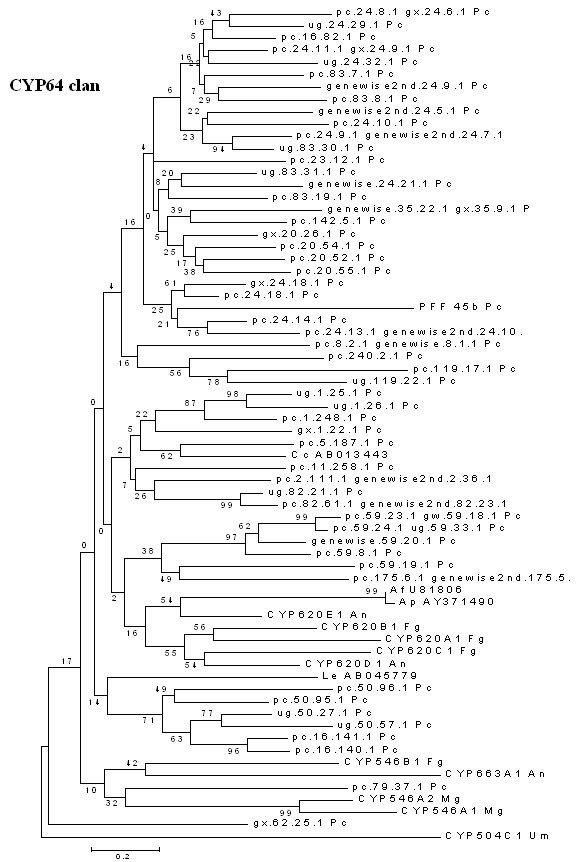
**Minimal evolution tree of the fungal CYP64 clan. **The unrooted phylogenetic tree is based on 69 P450 sequences (54 genes from *P. chrysosporium *and 15 genes from six other fungal species). Abbreviations: Af-*Aspergillus flavus *;An-*Aspergillus nidulans *; Ap-*Aspergillus parasiticus *; Cc-*Coprinus cinereus *;Fg-*Fusarium graminearum *; Le-*Lentinula edodes *; Mg-*Magnaporthe grisea *; Pc-*Phanerochaete chrysosporium; *Um-*Ustilago maydis. *

The CYP64 family, first identified in *Aspergillus *spp., was shown to include P450 proteins that catalyze various reactions involved in biosynthesis of aflatoxins and other such secondary metabolites in these species [35]. Genome-wide sequence similarity analysis and annotation of the *P. chrysosporium *P450ome revealed that 54 P450 genes fall under the CYP64 clan (Figure 8). The *P. chrysosporium *CYP64 family members are interspersed on the tree (as indicated by low bootstrap values on the critical branch nodes linking *P. chrysosporium *with other fungi) constructed based on the CYP64 clan genes from other fungal groups suggesting their multiple lineage or diversification. White rot fungi such as *P. chrysosporium *, which have a typical secondary metabolic switch in their growth/ biodegradation cycle, may have the hitherto uncharacterized role for its CYP64 clan proteins in secondary metabolism. Functional evaluation of one or more members from this group will bring into light the enzymatic or functional diversity of such a large number of CYP64-like genes in this fungus.

##### CYP505 clan

The fatty acid hydroxylase P450BM3 (CYP102) of the bacterium *Bacillus megaterium *, containing a P450 monooxygenase gene fused with a P450 reductase gene, was the first identified fusion protein member of the P450 superfamily [4]. Later, a similar fused P450 gene coding for fatty acid hydroxylase was identified from the fungus *Fusarium oxysporum *[18]. The latter, named P450foxy, shows 40.6% and 35.3% amino acid similarity in its P450 and reductase domains to the corresponding domains in the bacterial P450 fusion protein P450BM3. The current hypothesis suggests that such fused proteins are of eukaryotic origin and their occurrence in the prokaryotic (bacterial) cells is due to horizontal gene transfer [18].

**Figure 9:**
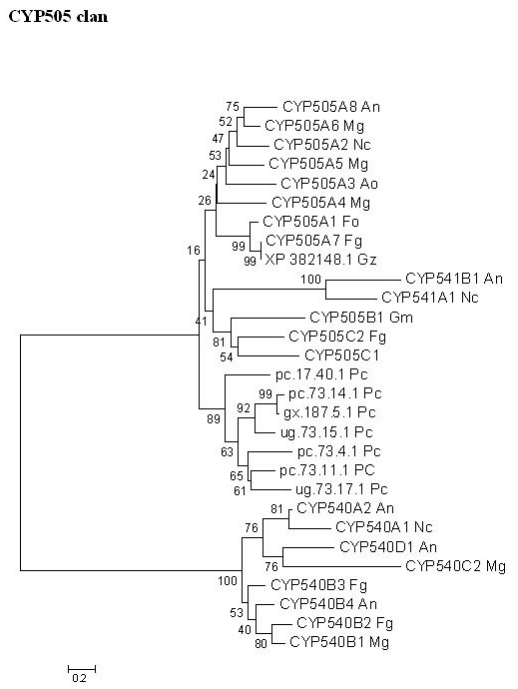
**Minimal evolution tree of the fungal CYP505 clan. **The unrooted phylogentic tree was constructed using only the P450 portion of the fused protein (P450foxy). Abbreviations: An-*Aspergillus nidulans *; Ao-*Aspergillus oryzae *; Fg-*Fusarium graminearum *; Fo-*Fusarium oxysporum *; Gm-*Gibberella moniliformis *; Gz-*Gibberella zeae *; Mg-*Magnaporthe grisea *; Nc-*Neurospora crassa *; Pc-*Phanerochaete chrysosporium *.

**Figure 10:**
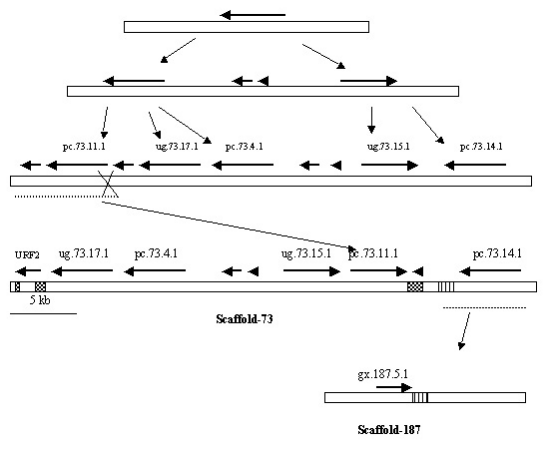
**Proposed microevolution of six of the P450foxy proteins in *P. chrysosporium *by gene duplication and translocation events. **Position and orientation of the genes are indicated with thick arrows above the horizontal scaffold bar. One or more initial tandem duplications were followed by translocation and ectopic integration (as indicated by thin arrows) of the fused genes leading to the present day organization. The regions of high sequence similarity on the scaffolds are shown in the same hatching.

Interestingly, there are 7-fused P450foxy-like genes in the *P. chrysosporium *genome located on 3 different scaffolds. These seven P450s form a separate cluster from the known P450foxy proteins of other fungi (ascomycetes) on the minimal evolution tree (Figure 9). There are two distinct clusters among the ascomycetous fungi (Figure 9), which appear to be lineages of two ancestral fused proteins based on their phylogenetic distance; of these, one clusters more closely with the *P. chrysosporium *fusion proteins. The white rot fusion proteins form three sub-groups within their phylogenetic branch separated by reasonably high bootstrap values (89% separating subgroup 1 from subgroups 2 and 3, and 63% separating subgroup 2 from subgroup 3), indicating considerable divergence among these proteins. Nevertheless, these fused proteins, while forming three sub-groups within their phylogenetic branch, show high conservation of functional domain sequences and intron/exon organization, suggesting the involvement of gene duplication and translocation events in their formation. There are multiple lines of evidence to support this argument. First, as discussed earlier, five out of the seven fused proteins are placed within a distance of 43 kb region on scaffold 73. Second, the conserved intron positioning in all agrees perfectly with their common grouping on the tree (Figure 9). Third, multiple regions of high sequence similarity in the flanking regions around these genes have been identified using the BLAST program. For instance, when the 43 kb region of the scaffold 73 was blasted against itself, a near perfect inverse duplication region of 2 kb was identified; the region from base number 32876 to 34876 is identical to the regions spanning base numbers 27 to 801 and 2134 to 3348 on scaffold 73. This explains the awkward position of the pc.73.11.1 gene between the closely related genes ug.73.15.1 and pc.73.14.1 on this scaffold and indicates a duplicative inversion event (Figure 10). Similarly, there is high sequence similarity (E-value = 0) between the 1000 bp non-coding flanking region downstream of the gx.187.5.1 gene, spanning base numbers 24674 to 25673 on scaffold 187, and the 983 bp non-coding region upstream of gene pc.73.1.1 spanning base numbers 36423 to 37405 on scaffold 73. This points to an ectopic insertion of the gx.187.5.1 on scaffold 187 after duplication on scaffold 73. This finding of an extraordinary level of sequence identity for non-coding DNA regions provides further evidence to the proposed involvement of gene duplication and translocation events.

Many questions regarding the origin and distribution of these fused P450 proteins in fungi remain unanswered. One can conclude from the phylogenetic tree (Figure 9) that more than one original fusion event happened in the ancestral fungus either before the ascomycetous-basidiomycetous split approximately 400 million years ago [36], or a second fusion event happened in ascomycetous fungi immediately after these two groups split. Either way, during the course of evolution, these proteins have diversified further, possibly due to more gene fusions or due to gene duplications (Figure 10). Assuming that the ancestral fungus carried two fusion proteins, the question as to what happened to the other gene lineage in basidiomycetous fungi arises; has it been lost immediately after these two groups split without further diversification or was it never there and the second lineage in the ascomycetous fungi originated after these groups had separated? However, there is one complication to this argument; if P450foxy proteins predate the ascomycetous-basidiomycetous split, then why are these proteins missing entirely in archiascomycotina and hemiascomycotina (fission and budding yeasts)? This is an open question and may be answered by analyzing the genomes of vesicular-arbuscular mycorrhizas (VAM) or chitrids, which predate these two groups' split [36]. The third possible reason for occurrence of these fused proteins in *P. chrysosporium *could be horizontal gene transfer from one or more of the ascomycetous fungi. When the *P. chrysosporium *P450foxy proteins were compared to the recently completed whole genome shotgun sequences of other basidiomycetes *Coprinus cinereus *, *Cryptococcus neoformans *, and *Ustilago maydis *, no P450 fused protein homologues were found indicating the unique presence of these proteins in *P. chrysosporium*, a member of the wood-rooting group of basidiomycetes. However, as more fungal genomes become available, especially from the basidiomycetous group, it will be clear if these fusion proteins are actually unique in *P. chrysosporium *(or wood-rotting basidiomycetes subgroup) among basidiomycetous fungi. Nevertheless, it appears that fused proteins are predominantly present across ascomycetous fungi, and their presumed exceptional occurrence in the basidiomycete *P. chrysosporium *could possibly be a result of a horizontal gene transfer, an event otherwise rare in fungal genome evolution [37,23,24]. Looking at the gene organization and flanking sequence homology of these P450 genes in *P. chrysosporium *in Figures 9 and 10, and their distribution on the genome, it appears that six of the seven genes have branched out from a single progenitor gene, while the origin of the seventh gene pc.17.40.1 remains unclear and could possibly be a result of independent transfer. Role of intragenomic duplication event in the origin of pc.17.40.1 is ruled out based on the fact that there is no flanking sequence similarity between this and the other six genes. Furthermore, *P. chrysosporium *has the largest contingent (7 member genes) of P450foxy proteins (CYP505) among the fungi (3–5 member genes) containing this family of proteins. These observations collectively point to a rapid evolution of this P450 fusion proteins family (CYP505) in *P. chrysosporium*, possibly to meet the metabolic demand for fatty acid hydroxylation in the ecological niches of this fungus.

##### Clan-level relationships of other *P. chrysosporium *P450 families

**Figure 11:**
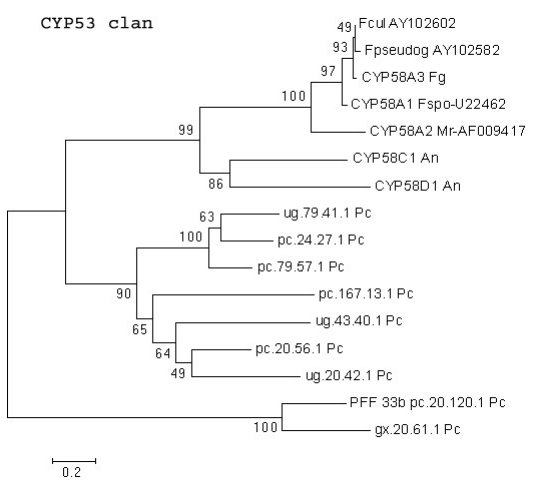
**Minimal evolution tree of the fungal CYP53 clan. **Nine *P. chrysosporium *CYP58 homologues were compared with the known CYP58 sequences from other fungal species. Abbreviations: An-*Aspergillus nidulans *; Fcul-*Fusarium culmorum *; Fg-*Fusarium graminearum *; Fpseudog-*Fusarium pseudograminearum *; Fs-*Fusarium sporotrichioides *; Mr-*Myrothecium roridum *; Pc-*Phanerochaete chrysosporium *.

The *P. chrysosporium *genome contains one member of the benzoate 4-hydroxylase family (CYP53) and nine members homologous to CYP58, both clustering under a common family on the *P. chrysosporium *tree (Figure 1) and groupable into the CYP53 clan . Presence of a single CYP53 protein in *P. chrysosporium *contrasts with the multiple CYP53 proteins detected in ascomycetous fungi. A total of 170 sequences (including CYP53 and CYP58) that are groupable into 4 sub-classes (A, B, C and D) have been assigned to the CYP53 clan in different ascomycetous fungi . The *P. chrysosporium *CYP53 protein is groupable under the B sub-class. The CYP58 family proteins have been shown to be involved in the synthesis of a group of secondary metabolites (trichothecene mycotoxins) in *Fusarium *spp. [38]. These proteins are grouped under the CYP53 clan sub-class C. However, the *P. chrysosporium *CYP58 homologues form a cluster separate from other fungal CYP58 proteins on the minimal evolution tree (Figure 11). Hence, we cannot rule out the possibility that these CYP58 structural homologues have evolved as functional variants, acquiring both CYP53 and CYP58 activities.

**Figure 12:**
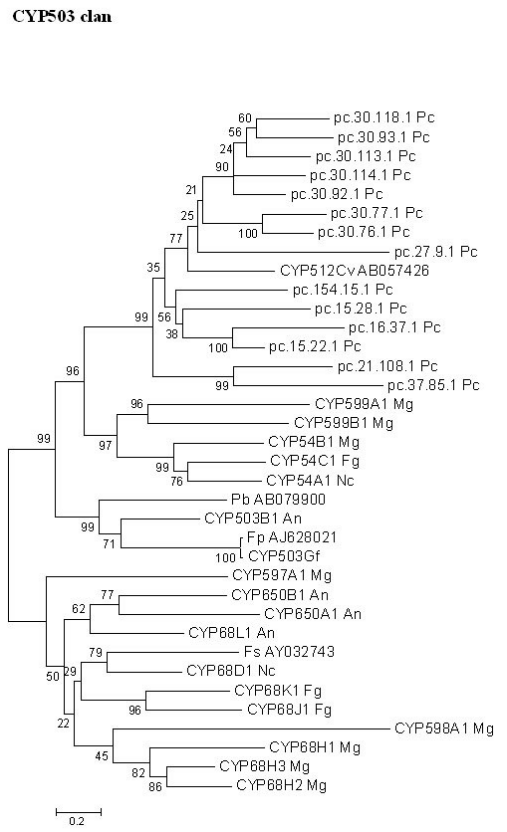
**Minimal evolution tree of the fungal CYP503 clan. **Fourteen *P. chrysosporium *P450 genes were compared with the known homologous P450 proteins from other fungi. Abbreviations: An-*Aspergillus nidulans *; Cv-*Coriolus versicolor *; Fg-*Fusarium graminearum *; Fp-*Fusarium proliferatum *; Fs-*Fusarium sporotrichioides *; Gf-*Gibberella fujikuroi *; Mg-*Magnaporthe grisea *; Nc-*Neurospora crassa *; Pc-*Phanerochaete chrysosporium; *Pb-*Phoma betae *.

Phylogenetic analysis based on the fungal CYP503 clan revealed that 12 of the *P. chrysosporium *P450 genes show close relatedness to the CYP512A1 gene from another lignin-degrading basidiomycete, *Coriolus versicolor *, but are distant from the CYP54 family and other CYP503 clan proteins from ascomycetous fungi, as shown by a high bootstrap value of 96% (Figure 12). The CYP503 family of P450 proteins originally found in *Gibberella fujikuroi *encode for multifunctional *ent *-kaurene oxidases that catalyze oxidation steps in the gibberellin biosynthesis of the plant growth hormone gibberellin, a secondary metabolite in this fungus. This is suggestive of a possible role of *P. chrysosporium *CYP503 clan genes in secondary metabolism.

**Figure 13:**
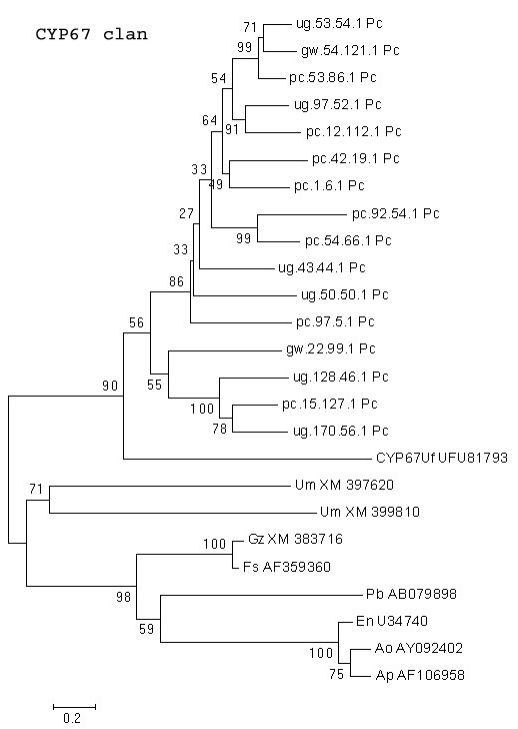
**Minimal evolution tree of the fungal CYP67 clan. **P450 genes from *P. chrysosporium *were compared with the known homologous P450 proteins from other fungal species. Abbreviations: An-*Aspergillus nidulans *; Ao-*Aspergillus ochraceoroseus *; En-*Emericella nidulans *; Fg-*Fusarium graminearum *; Fs-*Fusarium sporotrichioides *; Mg-*Magnaporthe grisea *; Nc-*Neurospora crassa *; Pc-*Phanerochaete chrysosporium *; Pb-*Phoma betae; *Uf-*Uromyces fabae *.

Sixteen *P. chrysosporium *P450 genes relate to the CYP67 clan (Figure 13). The CYP67 family was originally constituted of plant-induced rust genes identified in the basidiomycetous fungus *Uromyces fabae *[39]. The diterpene aphidicolin synthesizing gene PbP450-1 from *Phoma betae *and the genes involved in sterigmatocystin biosynthesis in *Emericella nidulans *are also groupable in the same clan in addition to some P450s encoding secondary metabolic reactions in other ascomycetous fungi (Figure 13). This suggests that the sixteen *P. chrysosporium *homologues might as well be involved in similar reaction steps in the synthesis of its secondary metabolites.

**Figure 14:**
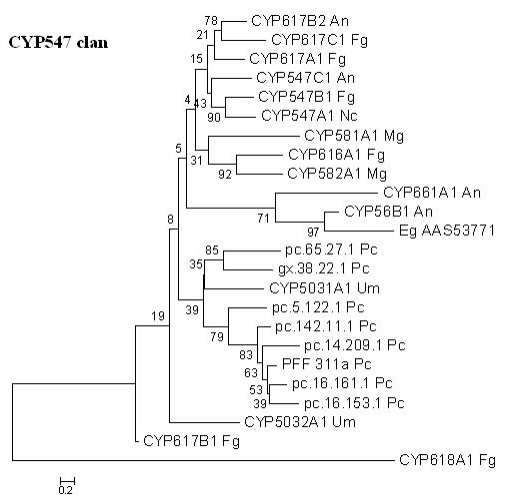
**Minimal evolution tree of the fungal CYP547 clan. **Eight P450 genes from *P. chrysosporium *showing homology with three P450 families, CYP547, CYP617, and CYP5031, were compared with homologous P450 sequences from other fungal species and were found to qualify as members of the CYP547 clan. Abbreviations: An-*Aspergillus nidulans *; Eg-*Eremothecium gossypii *; Fg-*Fusarium graminearum *; Mg-*Magnaporthe grisea *; Nc-*Neurospora crassa *; Pc-*Phanerochaete chrysosporium; *Um-*Ustilago maydis. *

Two *P. chrysosporium *P450 families, CYP617 and CYP5031, are groupable under the CYP547 clan. While the CYP547 clan proteins occur in all the higher ascomycetous fungi studied so far, two such proteins (CYP5031A1 and CYP5032A1) have also been identified recently in the basidiomycetous fungus *U. maydis *, of which CYP5031A1 shows closest relatedness (35% bootstrap value) to the two *P. chrysosporium *P450 proteins (Figure 14). On the other hand, the six other *P. chrysosporium *P450 proteins (pc.14.209.1, pc.16.161.1, PFF 311a, pc.142.11.1, pc.16.153.1 and pc.5.122.1), while showing highest BLAST homology to the CYP617 family of proteins, form a distinct group on the phylogenetic tree with a bootstrap value of 79% (Figure 14). It is possible that these six genes represent a unique family of P450 proteins, hitherto unidentified in other fungi. The same is true in the case of seven genes from the CYP614/534 clan and one gene (pc.96.21.1) from the CYP62 family of *P. chrysosporium *, which form independent clusters on the phylogenetic tree (Figures not shown).

Based on the clan-level comparison, it is evident that, although the *P. chrysosporium *P450ome has member counterparts among different fungal groups, in most cases, their independent clustering suggests significant sequence diversification and likely unique functionality. Such diversification emphasizes the need for assigning new family names to the *P. chrysosporium *P450s.

## Conclusion

The recognized extraordinary catalytic diversity of the white rot fungus *P. chrysosporium *correlates with its enormous P450 repertoire (P450ome), which is one of the largest among lower eukaryotes. Our structural and phylogenetic analyses of the P450ome, meant to understand the genesis of such a large number of P450 genes and facilitate their classification/nomenclature, have provided important clues to the evolution of the enormous catalytic diversity in this fungus. Considering the fact that certain P450 families (such as CYP64) have diversified more extensively than others, it appears that the *P. chrysosporium *P450ome has evolved in specific directions to meet the metabolic demand in its environmental niches. While the ancestral genes like CYP51 and CYP61 have remained unchanged, possibly due to their minimal role in *P. chrysosporium *(and other saprophytic fungi) versus that in their parasitic cousins such as pathogenic Aspergilli, other P450 gene families with suggestive roles in secondary metabolism (such as CYP64) have evolved as multigene families and even exist as gene clusters in this fungus. Such family-specific evolution was warranted presumably due to an extensive demand for generation of a broad range of metabolites in the secondary metabolic switch required for degradation of complex natural substrates such as lignin. The presented analysis indicates that the progenitor P450 families, originally acquired as a part of vertically descending P450ome, have diversified rapidly via multiple genetic mechanisms such as tandem duplications, translocations, mutations, and possibly gene fusions, to give rise to majority of the multigene families in the *P. chrysosporium *P450ome. Consequently, this structural diversification within individual multigene P450 families, as experimentally demonstrated in the case of CYP63 family in our studies, seems to have led to the acquisition of novel functions hitherto unseen in their ancestral counterparts (CYP52 genes of yeasts in this case). In addition, our experimental evidence for the presence of alternatively spliced variants in the *P. chrysosporium *P450 transcriptome further explains the evolution of expanded substrate diversity in this organism. The *P. chrysosporium *P450ome forms a model to investigate extrapolation of the evolved P450 gene diversity to the known vast biodegradation potential and will help design the future functional studies to understand the individual P450 gene functions in order to dissect the P450 functional diversity in white rot fungi.

## Methods

### Fungal cultures and cDNA cloning

*Phanerochaete chrysosporium *strain BKM-F-1767 (ATCC 24725) was grown as shaken cultures for 4 days in defined low nitrogen (LN) medium (2.4 mM N, 1% glucose) as described previously [14]. Total RNA (500 ng) was isolated from frozen fungal mycelia using the TRI Reagent kit (Molecular Research Center, Cincinnati, OH, USA) and a cDNA pool was generated using the SMART™ PCR cDNA synthesis kit (Clontech, Palo Alto, CA, USA) per the manufacturer's instructions. Briefly, first-strand cDNA synthesis step was carried out in a 10 μl reaction volume using 200 units of MMLV reverse transcriptase and 1 μM each of the SMART III Oligonucleotide and the CDS III/3' PCR Primer, at 42°C for 1 hour. One-fifth of the first-strand reaction (2 μl) was then added to a 100 μl long distance (LD)-PCR reaction with 1 μM each of the 5' PCR Primer and the CDS III/3' PCR Primer, for the synthesis of a double-stranded (ds) cDNA pool. Amplification parameters included initial denaturation at 95°C for 1 min., followed by a two step-PCR protocol involving use of 95°C for 1 min. and 68°C for 6 min., for 24 cycles. Quality of the ds cDNA amplified was analyzed on a 1.1% Agarose/EtBr gel and the product was quantified using spectrophotometer. Gene-specific cDNA isolation involved use of 100 ng of this cDNA mix as the template, in conjunction with an appropriate pair of gene-specific primers listed in Table 3. The gene-specific cDNA synthesis reaction contained 100 ng each of the forward and the reverse primer in a 50 μl PCR reaction volume and the amplification included 37 cycles, each involving denaturation at 95°C for 30 sec, annealing at 60°C for 30 sec, and extension at 72°C for 1 min.

The cDNA amplicons generated were cloned using 2.1-TOPO vector (Invitrogen, Carlsbad, CA, USA) per the manufacturer's instructions. The recombinant plasmid DNA for amplicon sequencing was isolated and purified using QIAprep Spin Miniprep kit (Qiagen, Valencia, CA) per the manufacturer's specifications. The DNA sequencing was performed at the university's DNA core facility. The cloned cDNA sequences generated in this study have been submitted to the GenBank under the accession numbers-AY835607 (*pc*-2), AY321373 (*pc*-4), AY321374 (*pc*-5), AY835606 (*pc*-6) and AY835608 (*pc-foxy*1).

### Sequence alignments and phylogenetic analysis

The *P. chrysosporium *P450 sequences used in the phylogenetic analysis were retrieved from the website  of the Joint Genome Institute of US Department of Energy- (US-DOE) and from the P450 website . The deduced amino acid sequence for a given P450 was compared from the above two sources and a sequence with the longest aa stretch was selected. However, the gene number assigned in the published white rot genome was retained for uniformity and convenience. For the cloned cDNAs, Gene Runner program (version 3.05, Hastings Software, Inc. Hastings, NY, USA) was used to extract and analyze the corresponding gene sequences from the genome, design the primers for RT-PCR amplification, and deduce the amino acid sequences. P450 sequences for other fungi were obtained from the NCBI GenBank database and the P450 website. Sequences were aligned using the CLUSTALW program at the EMBL-EBI website . Alignment of the 126 sequences of the *P. chrysosporium *P450ome was generated using the following customized parameters that varied from the default parameters- Matrix-BLOSUM (Henikoff), and Gap Open Penalty -1. All the other multiple alignments were generated using the default alignment parameters including the Matrix-GONNET 250 and Gap Open Penalty -10. Phylogenetic trees were constructed using the MEGA 2.1 software  [40]. The minimal evolution trees (Figures 5 to 10 and 11 to 14) were generated by heuristic search using the Close-Neighbor-Interchange (CNI) algorithm, with the Neighbor-Joining tree serving as the temporary tree. The topological distance (dT) was set at 2 for searching the minimal evolution tree. The *P. chrysosporium *P450ome (Figure 1) was constructed using the Unweighted Pair Group Method with Arithmatic Mean (UPGMA) method with gamma distance model. The alignment gaps and missing data sites were deleted and a bootstrap value based on 1000 replications was set for all the phylogenetic trees generated in this study. Protein sequences used for constructing the phylogenetic trees were obtained either from the GenBank (those shown with accession numbers) or from the P450 web site  (those with preassigned CYP names).

## Authors' contributions

HD performed the experiments, the analysis of the data, and the manuscript preparation. RC was involved in the phylogenetic analysis and interpretation of the evolutionary aspects of the paper. JSY who conceived of the study, was involved in its design and overall coordination, and helped in data interpretation and preparation of the manuscript. All authors have read and approved the final manuscript.

## Supplementary Material

Additional file 1 Click here for file

Additional file 2 Click here for file

Additional file 3 Click here for file
