# Supplementary material for: Genome-wide structural and evolutionary analysis of the P450 monooxygenase genes (P450ome) in the white rot fungus Phanerochaete chrysosporium : Evidence for gene duplications and extensive gene clustering
Source: BMC Genomics. 2005 Jun 14;6:92. doi: 10.1186/1471-2164-6-92 (PMC1184071; doi:10.1186/1471-2164-6-92)
Supplement: Additional file 1 [file 1471-2164-6-92-s1.doc]

**Table 1: Gene characteristics of the *P. chrysosporium*** P450 proteins

| **Gene** | **Family** | **# Exons** | **Exon size (bp)** | **Intron size (bp)** | **Protein (aa)** |
| --- | --- | --- | --- | --- | --- |
| pc.154.15.1 | CYP503 | 10 | 84-633 | 50-64 | 647 |
| pc.21.108.1 | CYP503 | 8 | 84-416 | 50-62 | 472 |
| pc.30.114.1 | CYP503 | 10 | 84-244 | 49-64 | 509 |
| pc.30.113.1 | CYP503 | 10 | 84-244 | 48-63 | 514 |
| pc.30.118.1 | CYP503 | 9 | 58-244 | 53-65 | 470 |
| gx.15.10.1 | CYP503 | 11 | 78-244 | 51-72 | 495 |
| gx.30.46.1 | CYP503 | 9 | 84-244 | 49-65 | 470 |
| pc.15.28.1 | CYP503 | 7 | 114-341 | 48-64 | 447 |
| pc.16.37.1 | CYP503 | 10 | 84-241 | 48-58 | 468 |
| pc.27.9.1 | CYP503 | 10 | 84-259 | 48-85 | 484 |
| pc.30.76.1 | CYP503 | 9 | 84-229 | 49-63 | 465 |
| pc.30.77.1 | CYP503 | 9 | 88-232 | 51-83 | 460 |
| pc.30.93.1 | CYP503 | 9 | 84-232 | 49-62 | 471 |
| pc.37.85.1 | CYP503 | 10 | 84-247 | 48-65 | 471 |
| gx.187.5.1 | CYP505 | 15 | 34-233 | 50-113 | 624 |
| pc.17.40.1 | CYP505 | 18 | 22-828 | 47-61 | 1058 |
| pc.73.14.1 | CYP505 | 21 | 34-758 | 49-103 | 1060 |
| pc.73.4.1 | CYP505 | 21 | 19-758 | 50-74 | 1055 |
| ug.73.15.1 | CYP505 | 20 | 26-758 | 46-103 | 1064 |
| ug.73.16.1 | CYP505 | 23 | 19-755 | 50-394 | 1111 |
| ug.73.17.1 | CYP505 | 22 | 19-758 | 46-66 | 1060 |
| ug.2.6.1 | CYP51 | 7 | 60-565 | 47-55 | 550 |
| *pc*-1 | CYP63 | 15 | 13-394 | 46-58 | 596 |
| *pc*-2 | CYP63 | 14 | 15-462 | 48-57 | 600 |
| *pc*-3 | CYP63 | 14 | 13-462 | 50-64 | 603 |
| *pc*-4 | CYP63 | 11 | 56-391 | 48-110 | 588 |
| *pc*-5 | CYP63 | 10 | 40-294 | 46-117 | 598 |
| *pc*-6 | CYP63 | 10 | 42-292 | 49-123 | 571 |
| *pc*-7 | CYP63 | 7 | 73-384 | 54-100 | 594 |
| ug.1.19.1 | CYP53 | 7 | 53-427 | 45-231 | 522 |
| pc.167.13.1 | CYP58 | 8 | 39-615 | 50-70 | 496 |
| pc.24.27.1 | CYP58 | 7 | 100-303 | 54-124 | 483 |
| pc.79.57.1 | CYP58 | 8 | 29-428 | 38-59 | 496 |
| ug.20.42.1 | CYP58 | 7 | 76-312 | 53-121 | 470 |
| ug.20.43.1 /pc.20.56.1 | CYP58 | 6 | 76-382 | 51-110 | 475 |
| ug.43.40.1 | CYP58 | 8 | 29-382 | 41-67 | 486 |
| ug.79.41.1 | CYP58 | 8 | 29-309 | 50-67 | 485 |
| gx.20.61.1 | CYP58 | 7 | 78-417 | 26-335 | 429 |
| pc.20.120.1 | CYP58 | 7 | 45-370 | 36-227 | 355 |
| ug.1.19.1/pc.1.261.1 | CYP58 | 5 | 72-396 | 50-231 | 536 |
| pc.65.27.1 | CYP5031/547 | 8 | 66-514 | 49-269 | 507 |
| gx.38.22.1 | CYP5031/547 | 8 | 20-391 | 50-139 | 482 |
|  |  |  |  |  |  |
| ug.78.18.1 | CYP61 | 4 | 75-1048 | 54-63 | 514 |
| pc.96.21.1 | CYP62 | 6 | 142-368 | 48-454 | 465 |
| ug.119.22.1 | CYP64 | 11 | 6-300 | 41-93 | 534 |
| gw.59.18.1 | CYP64 | 8 | 76-283 | 49-169 | 491 |
| gw.59.20.1 | CYP64 | 9 | 76-256 | 46-157 | 475 |
| gw.8.1.1 | CYP64 | 11 | 17-273 | 49-121 | 483 |
| gx.1.22.1 | CYP64 | 12 | 14-271 | 50-61 | 484 |
| gx.175.5.1 | CYP64 | 12 | 54-203 | 49-63 | 492 |
| gx.2.36.1 | CYP64 | 10 | 61-325 | 50-130 | 501 |
| pc.20.52.1/gx.20.25.1 | CYP64 | 10 | 37-276 | 52-105 | 494 |
| gx.20.26.1 | CYP64 | 11 | 23-244 | 46-111 | 475 |
| gx.24.10.1 | CYP64 | 13 | 14-276 | 40-103 | 480 |
| gx.24.17.1 | CYP64 | 10 | 64-273 | 50-122 | 489 |
| gx.24.18.1 | CYP64 | 8 | 108-243 | 51-159 | 458 |
| gx.24.9.1 | CYP64 | 12 | 14-273 | 47-122 | 478 |
| gx.24.5.1 | CYP64 | 13 | 14-273 | 49-101 | 479 |
| gx.24.6.1 | CYP64 | 11 | 23-253 | 50-109 | 455 |
| gx.24.7.1 | CYP64 | 11 | 18-274 | 49-108 | 479 |
| gx.24.9.1 | CYP64 | 12 | 23-273 | 50-102 | 499 |
| gx.35.9.1 | CYP64 | 8 | 82-232 | 53-227 | 452 |
| gx.62.25.1 | CYP64 | 10 | 73-269 | 47-119 | 475 |
| gx.82.23.1 | CYP64 | 10 | 64-337 | 36-69 | 478 |
| pc.1.248.1 | CYP64 | 9 | 79-325 | 35-60 | 514 |
| pc.119.17.1 | CYP64 | 10 | 82-273 | 48-112 | 521 |
| pc.142.5.1 | CYP64 | 11 | 23-273 | 47-121 | 516 |
| pc.16.140.1 | CYP64 | 11 | 79-189 | 50-63 | 513 |
| pc.20.54.1 | CYP64 | 12 | 21-276 | 45-126 | 510 |
| pc.20.55.1 | CYP64 | 10 | 82-189 | 48-132 | 501 |
| pc.23.12.1 | CYP64 | 9 | 67-221 | 53-201 | 494 |
| pc.24.10.1 | CYP64 | 11 | 23-274 | 51-92 | 494 |
| pc.24.14.1 | CYP64 | 9 | 94-235 | 51-179 | 513 |
| pc.240.2.1 | CYP64 | 9 | 85-349 | 48-113 | 512 |
| pc.5.187.1 | CYP64 | 6 | 76-689 | 49-74 | 513 |
| pc.50.95.1 | CYP64 | 10 | 79-189 | 49-58 | 460 |
| pc.50.96.1 | CYP64 | 10 | 79-208 | 50-64 | 489 |
| pc.59.19.1 | CYP64 | 9 | 62-272 | 50-64 | 503 |
| pc.59.8.1 | CYP64 | 8 | 94-397 | 52-268 | 498 |
| pc.83.19.1 | CYP64 | 10 | 14-307 | 49-111 | 483 |
| pc.83.7.1 | CYP64 | 10 | 14-322 | 47-131 | 490 |
| pc.83.8.1 | CYP64 | 9 | 18-418 | 49-175 | 524 |
| ug.1.25.1 | CYP64 | 10 | 14-325 | 28-106 | 508 |
| ug.1.26.1 | CYP64 | 9 | 39-325 | 48-115 | 514 |
| pc.11.258.1/ug.11.23.1 | CYP64 | 10 | 58-335 | 21-96 | 509 |
| ug.24.29.1 | CYP64 | 12 | 23-253 | 50-108 | 516 |
| ug.24.32.1 | CYP64 | 12 | 23-276 | 49-92 | 519 |
| ug.50.27.1 | CYP64 | 11 | 79-190 | 47-60 | 518 |
| ug.59.33.1 | CYP64 | 9 | 87-323 | 48-88 | 532 |
| ug.82.21.1 | CYP64 | 10 | 67-337 | 44-74 | 510 |
| ug.83.30.1 | CYP64 | 10 | 14-313 | 47-103 | 499 |
| ug.83.31.1 | CYP64 | 12 | 14-263 | 49-136 | 506 |
| pc.79.37.1 | CYP64 | 8 | 46-260 | 46-127 | 421 |
| ug.50.57.1 | CYP64 | 7 | 41-350 | 52-85 | 451 |
| pc.16.82.1 | CYP64 | 7 | 23-229 | 48-110 | 352 |
| gx.24.21.1/pc.24.27.1 | CYP64 | 7 | 100-303 | 54-124 | 483 |
| pff-45b | CYP64 | 9 | 67-132 | 53-201 | 350 |
| pc.16.141.1/gx.16.62.1 | CYP64 | 11 | 67-190 | 50-63 | 483 |
|  |  |  |  |  |  |
| pc.92.54.1 | CYP67 | 6 | 90-316 | 35-82 | 481 |
| ug.50.50.1 | CYP67 | 8 | 63-358 | 50-92 | 554 |
| ug.97.52.1 | CYP67 | 10 | 24-367 | 55-69 | 557 |
| ug.170.56.1 | CYP67 | 8 | 48-411 | 50-59 | 556 |
| gw.22.99.1 | CYP67 | 8 | 78-385 | 49-108 | 477 |
| gw.42.13.1 | CYP67 | 7 | 82-328 | 51-167 | 513 |
| gw.54.12.1 | CYP67 | 8 | 78-340 | 39-91 | 528 |
| gw.97.3.1 | CYP67 | 7 | 78-328 | 47-135 | 495 |
| pc.1.6.1 | CYP67 | 7 | 78-410 | 51-74 | 529 |
| pc.15.127.1 | CYP67 | 7 | 78-498 | 48-56 | 540 |
| pc.53.86.1 | CYP67 | 8 | 78-367 | 50-105 | 519 |
| pc.54.66.1 | CYP67 | 6 | 90-550 | 49-88 | 583 |
| ug.128.46.1 | CYP67 | 10 | 27-382 | 50-109 | 545 |
| pc.12.112.1 | CYP67 | 11 | 104-756 | 44-57 | 560 |
| ug.43.44.1 | CYP67 | 9 | 78-409 | 47-342 | 568 |
| ug.53.54.1 | CYP67 | 10 | 24-370 | 46-71 | 549 |
| pff311a | CYP617/547 | 12 | 39-266 | 47-115 | 523 |
| pc.14.209.1 | CYP617/547 | 12 | 30-266 | 31-54 | 513 |
| pc.16.161.1 | CYP617/547 | 12 | 30-541 | 51-121 | 563 |
| pc.142.11.1 | CYP617/547 | 13 | 30-266 | 50-58 | 539 |
| pc.16.153.1 | CYP617/547 | 6 | 101-351 | 50-305 | 306 |
| pc.5.122.1 | CYP617/547 | 12 | 48-348 | 45-56 | 535 |
| pc.181.9.1 | CYP614/534 | 16 | 31-148 | 49-118 | 514 |
| gx.37.18.1 | CYP614/534 | 14 | 34-274 | 47-119 | 533 |
| pff77b | CYP614/534 | 14 | 31-296 | 48-117 | 509 |
| pc.181.12.1 | CYP614/534 | 12 | 34-267 | 51-166 | 528 |
| pc.37.84.1 | CYP614/534 | 15 | 27-154 | 48-120 | 509 |
| pc.81.19.1 | CYP614/534 | 5 | 117-356 | 47-620 | 547 |
| pc.81.21.1 | CYP614/534 | 11 | 31-484 | 51-106 | 525 |
