# Supplementary material for: Genome-wide structural and evolutionary analysis of the P450 monooxygenase genes (P450ome) in the white rot fungus Phanerochaete chrysosporium : Evidence for gene duplications and extensive gene clustering
Source: BMC Genomics. 2005 Jun 14;6:92. doi: 10.1186/1471-2164-6-92 (PMC1184071; doi:10.1186/1471-2164-6-92)
Supplement: Additional file 2 [file 1471-2164-6-92-s2.doc]

**Table 2: Tandem gene clusters in the *P. chrysosporium*** genome

| Cluster # | P450 family | JGI scaffold # | Gene | Coding strand orientation | Gene structure  conservation | Gene order |
| --- | --- | --- | --- | --- | --- | --- |
| 1 | CYP64 | 1 | pc.1.247.1 | - | Yes | 1 |
|  |  |  | pc.1.248.1 | - |  | 2 |
|  |  |  |  |  |  |  |
| 2 | CYP64 | 16 | pc.16.82.1 | + | No | 1 |
|  |  |  | pc.16.83.1 | + |  | 2 |
|  |  |  |  |  |  |  |
| 3 | CYP64 | 20 | genewise2nd.20.25.1 | + | No | 1 |
|  |  |  | genewise2nd.20.26.1 | + |  | 2 |
|  |  |  | pc.20.54.1 | + |  | 3 |
|  |  |  | pc.20.55.1 | + |  | 4 |
|  |  |  | ug.20.43.1 | - |  | 5 |
|  |  |  |  |  |  |  |
| 4 | CYP63 | 20 | ug.20.34.1 (*pc*-3) | - | Yes | 1 |
|  |  |  | ug.20.35.1(*pc*-2) | - |  | 2 |
|  |  |  | ug.20.36.1(*pc*-1) | - |  | 3 |
|  |  |  |  |  |  |  |
| 5 | CYP64 | 24 | ug.24.32.1 | + | No | 1 |
|  |  |  | ug.24.29.1 |  |  | 2 |
|  |  |  | genewise2nd.24.5.1 | + |  | 3 |
|  |  |  | genewise2nd.24.6.1 | + |  | 4 |
|  |  |  | genewise2nd.24.7.1 | + |  | 5 |
|  |  |  | pc.20.10.1 | - |  | 6 |
|  |  |  | genewise2nd.24.9.1 | - |  | 7 |
|  |  |  | genewise2nd.24.10.1 | + |  | 8 |
|  |  |  | pc.24.14.1 | - |  | 9 |
|  |  |  | genewise2nd.24.12.1 | - |  | 10 |
|  |  |  | pc.24.16.1 | + |  | 11 |
|  |  |  |  |  |  |  |
| 6 | CYP64 | 24 | pc.24.18.1 | + | No | 1 |
|  |  |  | genewise2nd.24.17.1 | + |  | 2 |
|  |  |  | genewise2nd.24.18.1 | + |  | 3 |
|  |  |  | genewise2nd.24.19.1 | + |  | 4 |
|  |  |  |  |  |  |  |
| 7 | CYP503 | 30 | pc.30.76.1 | + | Yes | 1 |
|  |  |  | pc.30.77.1 | + |  | 2 |
|  |  |  | pc.30.78.1 | + |  | 3 |
|  |  |  |  |  |  |  |
| 8 | CYP503 | 30 | genewise2nd.30.46.1 | + | Yes | 1 |
|  |  |  | pc.30.93.1 | + |  | 2 |
|  |  |  |  |  |  |  |
| 9 | CYP503 | 30 | pc.30.113.1 | - | Yes | 1 |
|  |  |  | pc.30.114.1 | - |  | 2 |
|  |  |  |  |  |  |  |
| 10 | CYP64 | 50 | pc.50.95.1 | + | Yes | 1 |
|  |  |  | genscan.50.42.1 | + |  | 2 |
|  |  |  |  |  |  |  |
| 11 | CYP67 | 53 | genscan.53.32.1 | + | Yes | 1 |
|  |  |  | pc.53.86.1 | + |  | 2 |
|  |  |  |  |  |  |  |
| 12 | CYP64 | 59 | pc.59.23.1 | + | Yes | 1 |
|  |  |  | pc.59.24.1 | + |  | 2 |
|  |  |  |  |  |  |  |
| 13 | CYP505 | 73 | ug.73.17.1 | - | Yes | 1 |
|  |  |  | pc.73.4.1 | - |  | 2 |
|  |  |  |  |  |  |  |
| 14 | CYP505 | 73 | ug.73.15.1 | + | No | 1 |
|  |  |  | ug.73.16.1 | + |  | 2 |
|  |  |  |  |  |  |  |
| 15 | CYP58 | 79 | ug.79.41.1 | + | Yes | 1 |
|  |  |  | pc.79.57.1 | + |  | 2 |
|  |  |  |  |  |  |  |
| 16 | CYP67 | 97 | pc.97.5.1 | - | No | 1 |
|  |  |  | ug.97.52.1 | - |  | 2 |
