# Supplementary material for: Genome-wide structural and evolutionary analysis of the P450 monooxygenase genes (P450ome) in the white rot fungus Phanerochaete chrysosporium : Evidence for gene duplications and extensive gene clustering
Source: BMC Genomics. 2005 Jun 14;6:92. doi: 10.1186/1471-2164-6-92 (PMC1184071; doi:10.1186/1471-2164-6-92)
Supplement: Additional file 3 [file 1471-2164-6-92-s3.doc]

Table 3: Oligonucleotide primers used for cDNA isolation in this study

| Target gene | Forward Primer | Reverse primer | Amplicon (bp) |
| --- | --- | --- | --- |
| *pc*-2 | **F5** [5’-CTGCCGCCATGTTGGTCTCCG- 3’] | **R10** [5’- GCAGCAGGGCACATCCACTAGG -3’] | 1842 |
| *pc*-4 | **F1** [5’-CCCTTCAACGTCCGCTTTAGCAC-3’] | **R2** [5’-CACCCTGCACGTACATCGTCTGG- 3’] | 430 |
| *pc*-5 | **F1** [5’-GCCTGCCAGACCATCCGTTTGC-3’] | **R2** [5’-CTCTCTCACCCACAAGCCACCC-3’] | 324 |
| *pc*-6 | **F1** [5’-GCCGATGCTCTCGACTTCCG-3’] | **R2** [5’-ACAAGGTCTGGAACGAGTCG-3’] | 330 |
| *pc-foxy1* | **F1** [5’-CATGCGCGCCAGACAGTACTCC-3’] | **R1** [5’-GCTATTCAAATACGTCGGTCGCG-3’] | 681 |
